# Supplementary material for: Global correlates of terrestrial and marine coverage by protected areas on islands
Source: Nat Commun. 2020 Sep 7;11:4438. doi: 10.1038/s41467-020-18293-z (PMC7477099; doi:10.1038/s41467-020-18293-z)
Supplement: Supplementary file 1 — Supplementary Information [file 41467_2020_18293_MOESM1_ESM.pdf]

Global correlates of terrestrial and marine coverage by protected areas on islands

David Mouillot et al.

Supplementary Figures

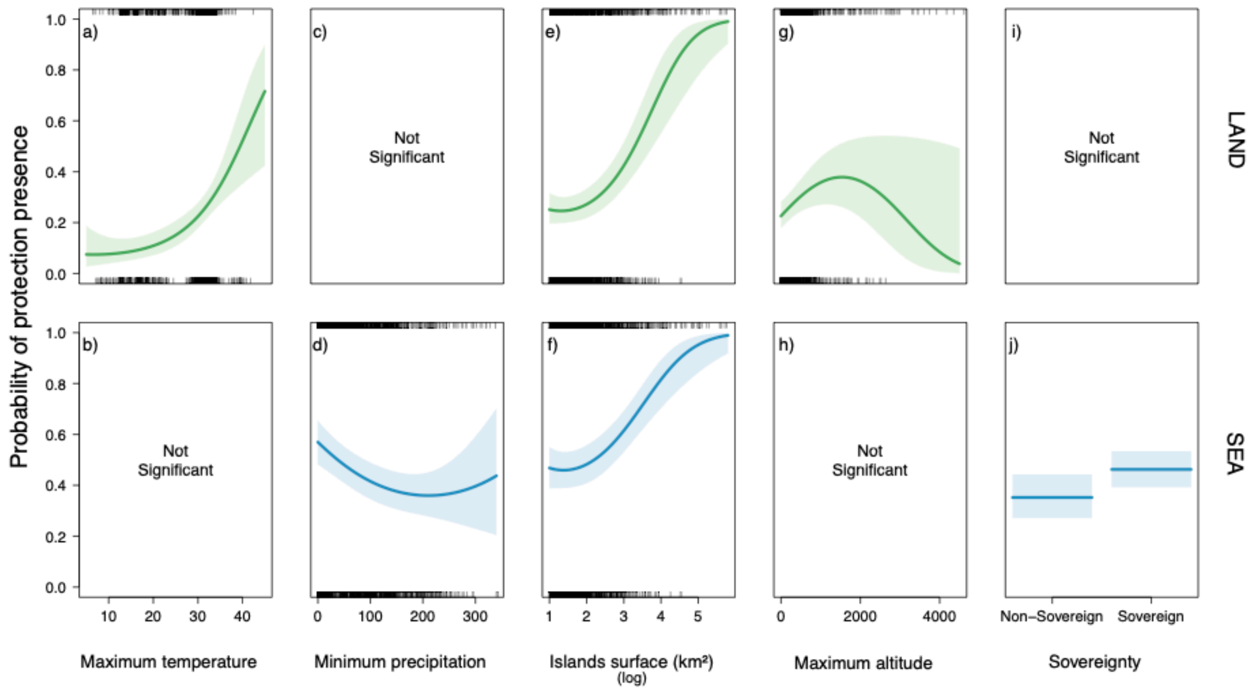

Supplementary Figure 1. Relationships between the presence of protected areas on islands and the secondary factors. Partial regression plots showing the influence of 5 secondary factors on the probability of having some protection coverage (presence/absence) on terrestrial (first raws, green curves) and marine (second raw, blue curves) areas of the 2,323 islands using the most parsimonious binomial generalized linear models. The colored shaded areas are the 95% confidence intervals of the relationships.

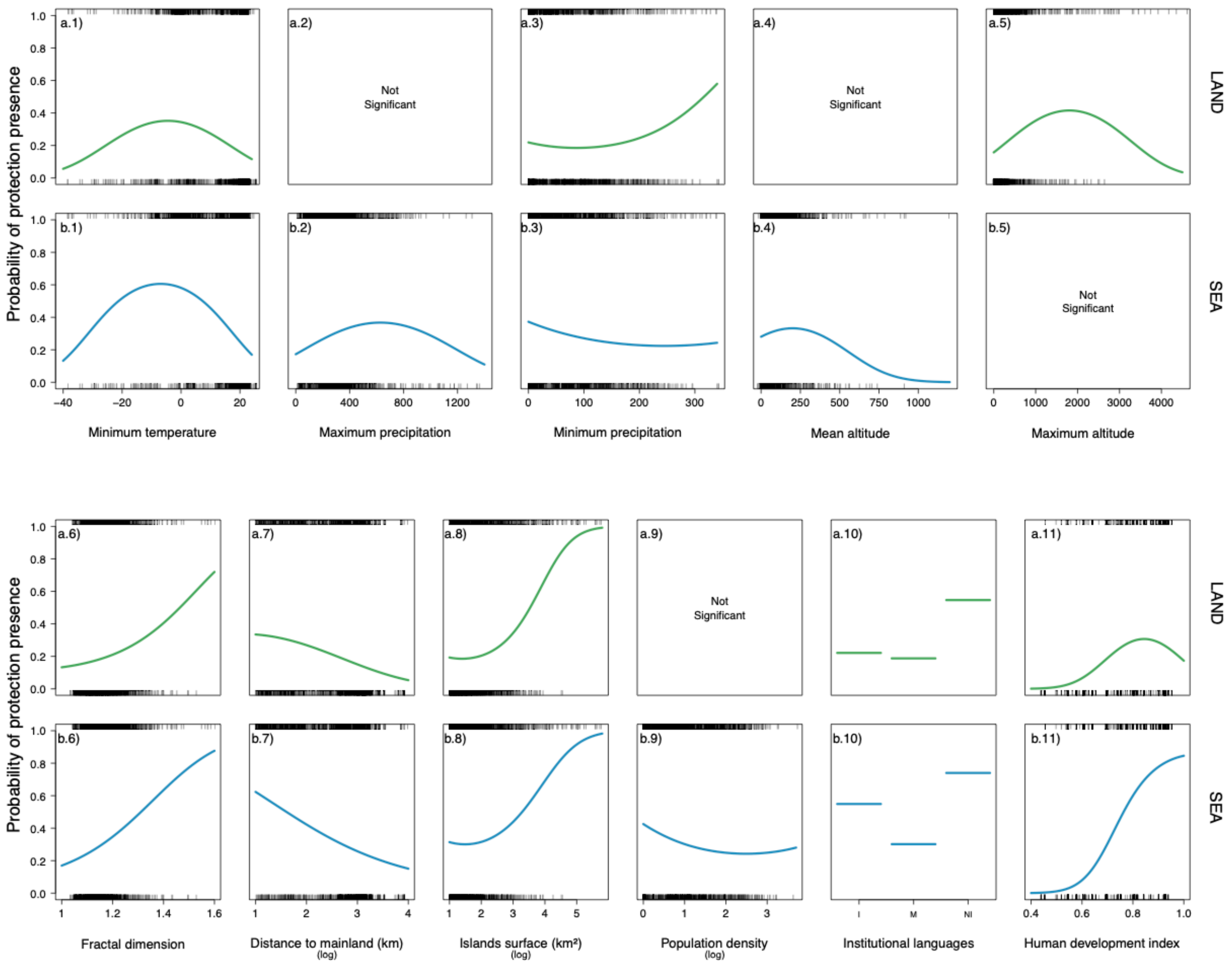

Supplementary Figure 2. Relationships between the presence of protected areas on islands and all selected factors. Partial regression plots showing the influence of all 16 factors on the probability of having some protection coverage (presence/absence) on terrestrial (first raws, green curves) and marine (second raws, blue curves) areas of the 2,323 islands the most parsimonious binomial generalized linear mixed-effects models with 'country' as a random effect.

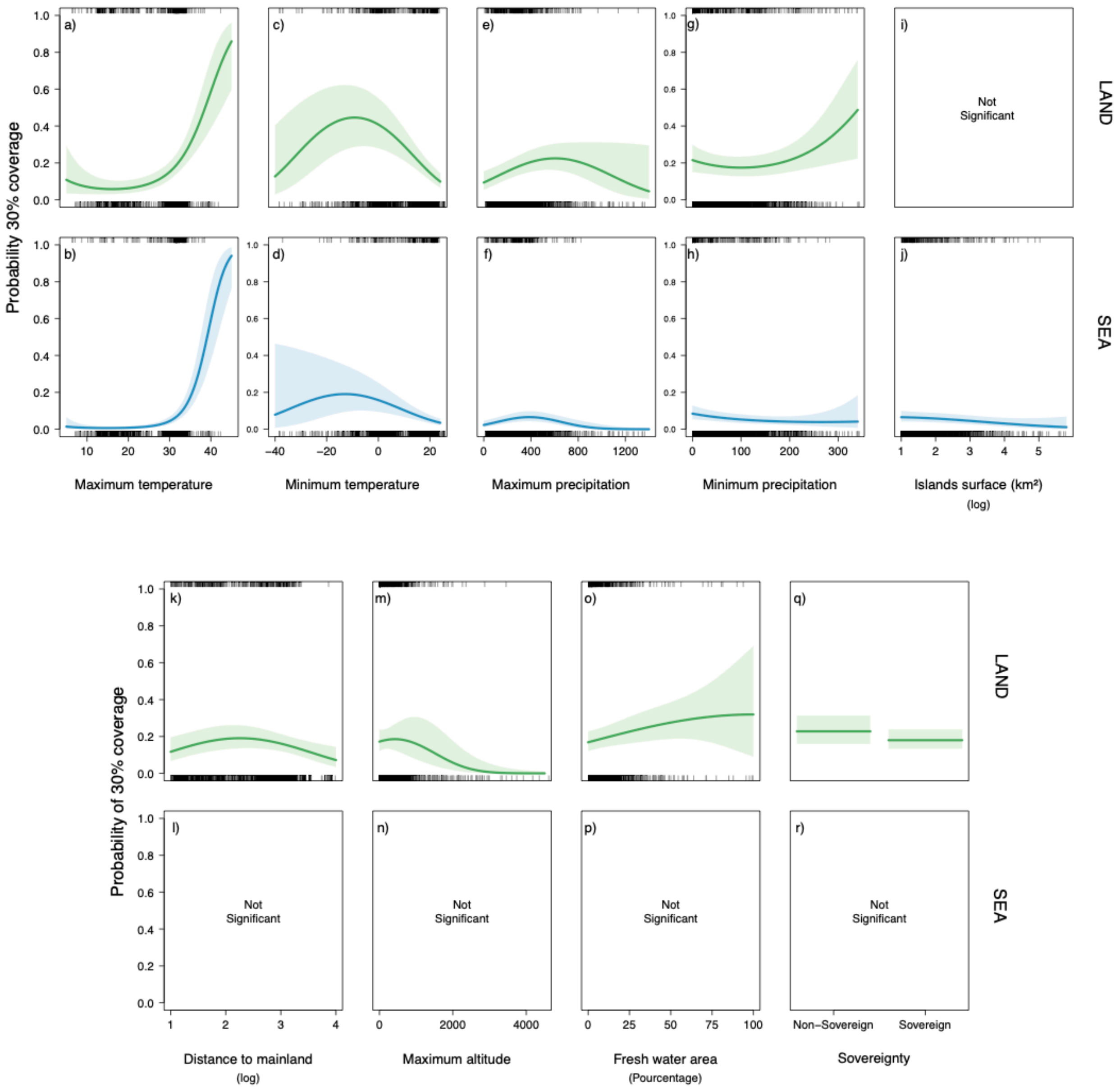

Supplementary Figure 3. Relationships between the achievement of 30% coverage by protected areas on islands and the secondary factors. Partial regression plots showing the influence of 9 secondary factors on the probability of achieving 2030 conservation targets (>30% coverage) on terrestrial (first raws) and marine (second raws) areas of the 2,323 islands using the most parsimonious binomial generalized linear models. The colored shaded areas are the 95% confidence intervals of the relationships.

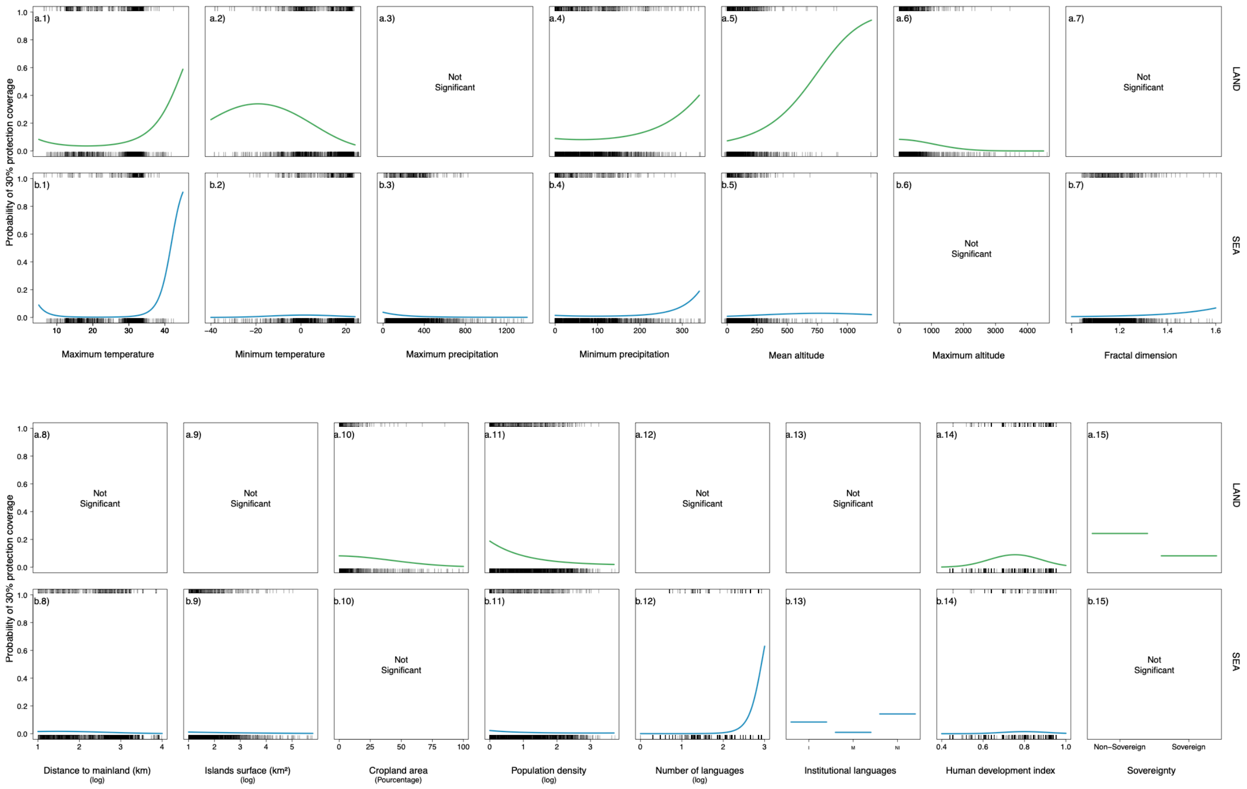

Supplementary Figure 4. Relationships between the achievement of 30% coverage by protected areas on islands and all selected factors. Partial regression plots showing the influence of all selected factors (AIC-based) on the probability of achieving 2030 conservation targets (>30% coverage) on terrestrial (first raws, green curves) and marine (second raws, blue curves) areas of the 2,323 islands using the most parsimonious binomial generalized linear mixed-effects models with ‘country’ as a random effect.

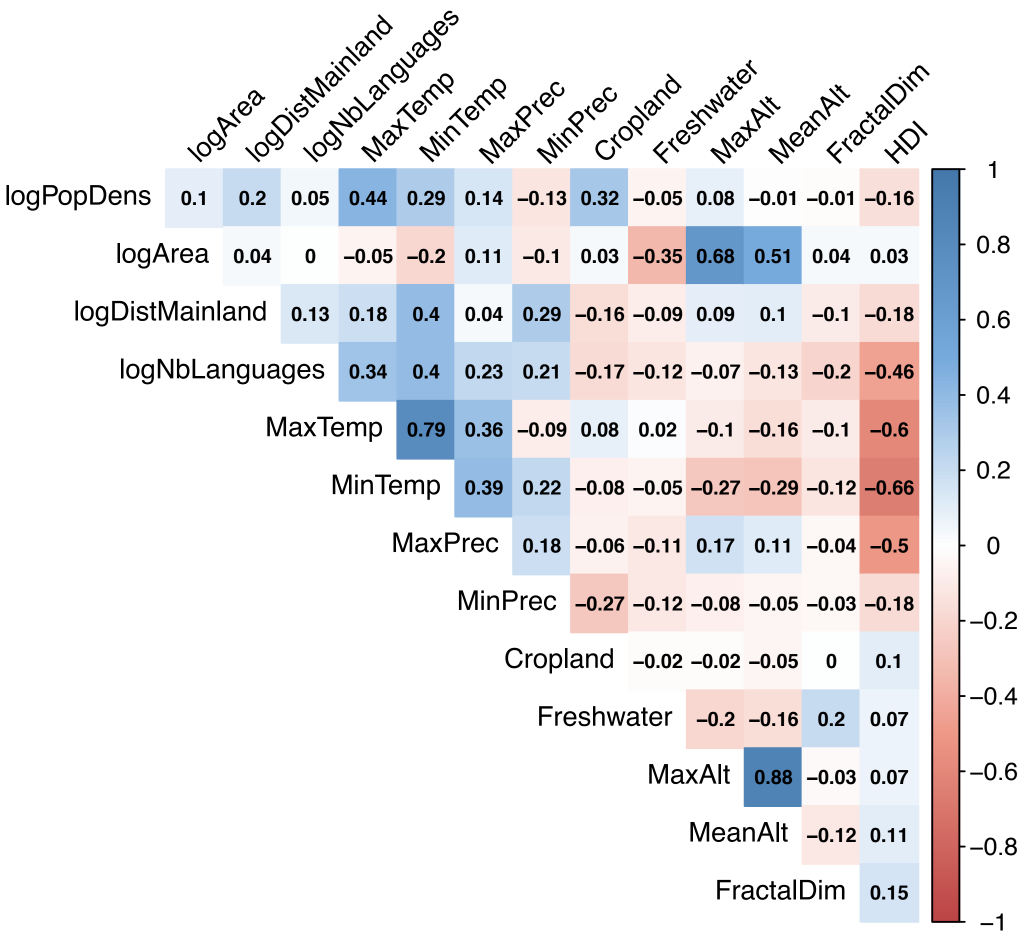

Supplementary Figure 5. Relationships between all pairs of factors. Correlogram showing the relationship (Pearson correlation coefficient) between all pairs of quantitative factors used in our analysis (see Methods).

Supplementary Tables

Supplementary Table 1. Results from binomial generalized linear mixed-effects models, with 'country' as a random effect, predicting the presence of protected areas and the achievement of 2030 targets (30% coverage on both terrestrial and marine areas) for 2,323 islands globally as a function of 16 factors (see Table 1 and Methods). df is the degree of freedom for each factor, AIC weight represents the importance of each factor in the best models and the F-value its influence on the predicted variable (^{NS}, Not significant, * $p<0.05$, ** $p<0.01$, *** $p<0.001$). Only factors retained in the most parsimonious models (according to a backward selection procedure based on AIC), in bold, are statistically tested.

		Presence of protection				30% of protection coverage			
		Terrestrial Protected Areas		Marine Protected Areas		Terrestrial Protected Areas		Marine Protected Areas	
	**df**	AIC Weight	F-value	AIC Weight	F-value	AIC Weight	F-value	AIC Weight	F-value
Maximum temperature	2	0.46		0.44		0.85	9.3***	1	7.4***
Minimum temperature	2	1	27.7***	1	24.4***	0.98	8.7***	0.92	3.9*
Maximum precipitation	2	0.13		0.54	2.4*	0.19		1	7.4***
Minimum precipitation	2	0.84	4.4**	0.60	2.6*	0.96	6.0**	0.84	4.6**
Surface area	2	1	33.3***	0.99	8.0***	0.48		0.58	2.3*
Distance to the mainland	2	1	12.9**	1	10.3***	0.33		0.94	4.6**
Maximum altitude	2	0.69	3.4*	0.31		0.94	6.6**	0.30	
Mean altitude	2	0.40		0.78	4.0*	0.70	1.3^{NS}	0.41	1.49^{NS}
Fractal dimension	2	1	7.2***	1	14.9***	0.19		0.93	5.2**
Cropland area	2	0.38		0.34		0.60	2.4*	0.33	
Freshwater area	2	0.16		0.19		0.29		0.30	
Population density	2	0.30		0.94	4.8**	1	34.2***	1	15.1***
Number of languages	2	0.46		0.27		0.33		1	4.9***
Institutional languages	2	0.79	8.5***	0.93	7.1**	0.58		0.91	3.1**
Sovereignty	2	0.28		0.38		0.85	7.3**	0.27	
Human Development Index	2	1	8.5***	1	13.8***	0.93	4.0*	0.63	4.4*
