# Supplementary material for: Global correlates of terrestrial and marine coverage by protected areas on islands
Source: Nat Commun. 2020 Sep 7;11:4438. doi: 10.1038/s41467-020-18293-z (PMC7477099; doi:10.1038/s41467-020-18293-z)
Supplement: Supplementary file 3 — Description of Additional Supplementary Files [file 41467_2020_18293_MOESM3_ESM.pdf]

**Description of Additional Supplementary Files**

**File Name**: Supplementary Data 1
**Description:** Raw data on 2,389 inhabited islands, containing 16 social and environmental factors.
