# Supplementary material for: Global correlates of terrestrial and marine coverage by protected areas on islands
Source: Nat Commun. 2020 Sep 7;11:4438. doi: 10.1038/s41467-020-18293-z (PMC7477099; doi:10.1038/s41467-020-18293-z)
Supplement: Supplementary file 5 — Reporting Summary [file 41467_2020_18293_MOESM5_ESM.pdf]

nature research

Corresponding author(s): David MOUILLOT

Last updated by author(s): 13/07/2020

Reporting Summary

Nature Research wishes to improve the reproducibility of the work that we publish. This form provides structure for consistency and transparency in reporting. For further information on Nature Research policies, see Authors & Referees and the Editorial Policy Checklist .

Statistics

For all statistical analyses, confirm that the following items are present in the figure legend, table legend, main text, or Methods section.

n/a	Confirmed	
☐	☒	The exact sample size (n) for each experimental group/condition, given as a discrete number and unit of measurement
☐	☒	A statement on whether measurements were taken from distinct samples or whether the same sample was measured repeatedly
☐	☒	The statistical test(s) used AND whether they are one- or two-sided<br>*Only common tests should be described solely by name; describe more complex techniques in the Methods section.*
☐	☒	A description of all covariates tested
☐	☒	A description of any assumptions or corrections, such as tests of normality and adjustment for multiple comparisons
☐	☒	A full description of the statistical parameters including central tendency (e.g. means) or other basic estimates (e.g. regression coefficient) AND variation (e.g. standard deviation) or associated estimates of uncertainty (e.g. confidence intervals)
☐	☒	For null hypothesis testing, the test statistic (e.g. F, t, r) with confidence intervals, effect sizes, degrees of freedom and P value noted<br>*Give P values as exact values whenever suitable.*
☒	☐	For Bayesian analysis, information on the choice of priors and Markov chain Monte Carlo settings
☒	☐	For hierarchical and complex designs, identification of the appropriate level for tests and full reporting of outcomes
☒	☐	Estimates of effect sizes (e.g. Cohen's d, Pearson's r), indicating how they were calculated

Our web collection on statistics for biologists contains articles on many of the points above.

Software and code

Policy information about availability of computer code

Data collection	No particular software was used to download the data.
Data analysis	No custom script developped only classical functions from ArcGIS 10.3 and R3.4

For manuscripts utilizing custom algorithms or software that are central to the research but not yet described in published literature, software must be made available to editors/reviewers. We strongly encourage code deposition in a community repository (e.g. GitHub). See the Nature Research guidelines for submitting code & software for further information.

Data

Policy information about availability of data

All manuscripts must include a data availability statement. This statement should provide the following information, where applicable:

- Accession codes, unique identifiers, or web links for publicly available datasets
- A list of figures that have associated raw data
- A description of any restrictions on data availability

All raw data are available in the Supplementary Dataset 1. GADM database of Global Administrative Areas, version 2.8. available at: www.gadm.org. Center for International Earth Science Information Network - CIESIN - Columbia University. 2016. Gridded Population of the World, Version 4 (GPWv4): Administrative Unit Center Points with Population Estimates. Palisades, NY: NASA Socioeconomic Data and Applications Center (SEDAC). http://dx.doi.org/10.7927/H4F47M2C. Accessed 09 10 2017. IUCN and UNEP-WCMC (2016), The World Database on Protected Areas (WDPA) [On-line], [09/2017]. Cambridge, UK: UNEP-WCMC. Available at: www.protectedplanet.net. The GEBCO_2014 Grid, version 20150318, http://www.gebco.net. Worldclim 2: New 1-km spatial resolution climate surfaces for global land areas. International Journal of Climatology. Available at: https://www.worldclim.org/data/bioclim.html. USGS Land Cover Institute: https://archive.usgs.gov/archive/sites/landcover.usgs.gov/globallandcover.html. Ethnologue Global Dataset (20th Edition). 2016 Human Development Report published by the United Nations of Development program (UNDP): http://www.hdr.undp.org.

Field-specific reporting

Please select the one below that is the best fit for your research. If you are not sure, read the appropriate sections before making your selection.

☐ Life sciences ☐ Behavioural & social sciences ☒ Ecological, evolutionary & environmental sciences

For a reference copy of the document with all sections, see nature.com/documents/nr-reporting-summary-flat.pdf

Ecological, evolutionary & environmental sciences study design

All studies must disclose on these points even when the disclosure is negative.

Study description	The goal was to assess the relative influence of climate, geography, habitat diversity, multiculturalism, governance capacity, and human footprint on the protection coverage of terrestrial and marine areas on 2,323 inhabited islands at the global scale. We use binomial GLM
Research sample	We extracted 2,503 island layers from the Global Administrative Areas - GADM v.2.8 (Global Administrative Areas 2016) spatial dataset
Sampling strategy	We considered all inhabited islands so no sampling strategy was applied
Data collection	All data were donwloaded from online resources by the second author Laure Velez
Timing and spatial scale	Global scale<br>Data from 2000 to 2019
Data exclusions	No data were excluded
Reproducibility	All analyses can be reproduced using raw data
Randomization	NA
Blinding	NA

Did the study involve field work? ☐ Yes ☒ No

Reporting for specific materials, systems and methods

We require information from authors about some types of materials, experimental systems and methods used in many studies. Here, indicate whether each material, system or method listed is relevant to your study. If you are not sure if a list item applies to your research, read the appropriate section before selecting a response.

Materials & experimental systems

n/a	Involved in the study	
☒	☐	Antibodies
☒	☐	Eukaryotic cell lines
☒	☐	Palaeontology
☒	☐	Animals and other organisms
☒	☐	Human research participants
☒	☐	Clinical data

Methods

n/a	Involved in the study	
☒	☐	ChIP-seq
☒	☐	Flow cytometry
☒	☐	MRI-based neuroimaging
